# Horizontal Transfer of a Subtilisin Gene from Plants into an Ancestor of the Plant Pathogenic Fungal Genus *Colletotrichum*

**DOI:** 10.1371/journal.pone.0059078

**Published:** 2013-03-15

**Authors:** Vinicio Danilo Armijos Jaramillo, Walter Alberto Vargas, Serenella Ana Sukno, Michael R. Thon

**Affiliations:** Centro Hispano-Luso de Investigaciones Agrarias, Departamento de Microbiología y Genética, Universidad de Salamanca, Villamayor, Spain; University of Ottawa, Canada

## Abstract

The genus *Colletotrichum* contains a large number of phytopathogenic fungi that produce enormous economic losses around the world. The effect of horizontal gene transfer (HGT) has not been studied yet in these organisms. Inter-Kingdom HGT into fungal genomes has been reported in the past but knowledge about the HGT between plants and fungi is particularly limited. We describe a gene in the genome of several species of the genus *Colletotrichum* with a strong resemblance to subtilisins typically found in plant genomes. Subtilisins are an important group of serine proteases, widely distributed in all of the kingdoms of life. Our hypothesis is that the gene was acquired by *Colletotrichum* spp. through (HGT) from plants to a *Colletotrichum* ancestor. We provide evidence to support this hypothesis in the form of phylogenetic analyses as well as a characterization of the similarity of the subtilisin at the primary, secondary and tertiary structural levels. The remarkable level of structural conservation of *Colletotrichum* plant-like subtilisin (CPLS) with plant subtilisins and the differences with the rest of *Colletotrichum* subtilisins suggests the possibility of molecular mimicry. Our phylogenetic analysis indicates that the HGT event would have occurred approximately 150–155 million years ago, after the divergence of the *Colletotrichum* lineage from other fungi. Gene expression analysis shows that the gene is modulated during the infection of maize by *C. graminicola* suggesting that it has a role in plant disease. Furthermore, the upregulation of the CPLS coincides with the downregulation of several plant genes encoding subtilisins. Based on the known roles of subtilisins in plant pathogenic fungi and the gene expression pattern that we observed, we postulate that the CPLSs have an important role in plant infection.

## Introduction

The genus *Colletotrichum* within the Ascomycetes includes a large number of phytopathogenic species that affect a wide range of crops worldwide [1], [2]. Species of this genus are the agents of anthracnose diseases that cause devastating yield losses in agriculture [3]. To achieve infection, *Colletotrichum* species employ a diversity of molecules such as effectors, kinases, hydrolytic enzymes and others [4]–[6]. Within the molecular arsenal of these organisms, the catalytic enzymes provide a wide range of tools to achieve successful host infection. One of the largest groups of catalytic enzymes is composed of serine proteases, a group of proteins that can be found in all kingdoms of life [7], [8]. These enzymes include endopeptidases and exopeptidases organized into 12 clans and 35 families according to the MEROPS peptidase database [9]. The MEROPS S8 family of subtilisins is especially important for the large number of proteins that it contains as well as its broad taxonomic distribution. The S8 family constitutes a heterogeneous group of proteins with a characteristic catalytic triad peptide (Asp, His and Ser), with no other structural resemblance attributable to all members of the family. The MEROPS database subdivides S8 subtilisins in two subfamilies, the real subtilisins as S8A and the S8B kexin subfamily that includes proprotein convertases [10], [11].

This extended family of enzymes presents a wide range of functions and their members are involved in a broad spectrum of metabolic processes in plants and fungi, with many members having roles in plant-microbe interactions (Table 1). An interesting group of proteins that belong to the S8A subtilisins are pathogenesis-related 7 (PR-7) proteins with roles in plant-pathogen interactions. PR proteins are defined as molecules that are induced in plants under pathological or related situations [12]. These proteins form a group with various chemical characteristics and biological functions. For that reason a standardization of the nomenclature was proposed, dividing the proteins by sequence similarity and enzymatic or biological activity [12]. One of those groups was named PR-7, represented by tomato P69 proteins [13]–[15] a group of proteins of family S8.

**Table 1:** Functions of subtilisin family members in plants and fungi.

Name	GenBank accession number	Organism	Function	Reference
**SbtM1 and** **SbtM3**	BAF95755, BAF95754	*Lotus japonicas*	A role in the development of arbuscular mycorrhiza	[81]
**Phytaspase**	ACT34764	*Nicotiana tabacum*	Involved in programmed cell death	[82]
**Gm-1**	Glyma18g48580.1 *	*Glycine max*	Contains the signal peptide GmSubPep that inducesdefense-related genes	[83]
**P69B and** **P69C**	CAA76725, CAA06412	*Solanum lycopersicum*	Pathogen-related proteins	[84], [85]
**Ara12**	AAN13181	*Arabidospsis thaliana*	Undefined. Probably involved in the development of plant	[86]
**Pr1**	ACZ28128	*Beauveria bassiana*	Virulence factor involved in the pathogenicity against insects	[87]
**Mp1**	AAD26255	*Magnaporthe poae*	Implicated in the infection process	[88]
**Ver112**	Q68GV9	*Lecanicillium psalliotae*	Nematicidal activity	[89]
**At1**	AAB62277	*Epichloe typhina*	Potential role in symbiosis	[90]

* Genome project accession number.

One of the most interesting characteristics of family S8 is their domain variability. The peptidase S8 domain (the most characteristic domain of this family, Pfam:PF00082) is usually found combined with various other domains. The domains typically found in family S8 are: PA (Pfam:PA02225), inhibitor I9 (Pfam:PF05922), alpha-1,3-glucanase (Pfam:PF03659), chitinase class II group (Pfam: PF00704), pectin lyase (SCOP:51133), cyclin domains (InterPro: IPR006670), DUF1034 (Domain of Unknown Function 1034, Pfam:PF06280), DUF1043(Pfam:PF06280), Cytochrome P450 (Pfam:PF00067), P domain (Pfam:PF01483) glyco_hydro_71 (Pfam:PF03659), glyco_hydro_18 (Pfam:PF00704), cyclin (InterPro:IPR006670), Pro-kuma_activ (SMART:SM00944), Sir2 (Pfam:PF02146) and sac_ganp (Pfam:PF03399). This domain combination could be identified in S8 subtilisins of animals, plants, bacteria or fungi [16].

The subtilisin S8 family represents an important group of proteases. Over 200 members have been identified in bacteria, archaeas, eukaryotes and viruses. In Arabidopsis 56 S8 family members have been identified, and 63 have been reported in maize [11]. In fungi, members of the S8 family are also abundant. At least four of the six subfamilies of subtilisins in the classification of [10] and [17] were found in different Ascomycota and Basidiomycota [16], [18]. *Colletotrichum higginsianum* contains thirty six subtilisins S8 and *C. graminicola* twelve. This family is apparently highly expanded in *C. higginsianum* compared with *C. graminicola* and other fungi [2].

Phylogenetic trees of the S8 family are, in general, congruent with the species tree [10], [16], [18] showing that subtilisins are predominantly transmitted vertically to descendents. To our knowledge, an event of horizontal gene transfer (HGT) has not been reported in eukaryotes for this family of proteins.

Cases of HGT were previously taken as isolated incidents and were not considered important, but now have gained enormous interest due to their consequences on species evolution. Numerous cases have been reported in recent years, especially in prokaryotes [19]–[23]. Reports of HGT events in eukaryotes are less abundant, congruent with the idea that HGT is rare in eukaryotic organisms. Barriers such differential intron processing, incompatible gene promoters, unpaired meiotic DNA, eukaryotic membranes, and alternative genetic codes may present obstacles for the horizontal transmission of genes [24], [25]. However, an increasing number of publications provide evidence of HGT in eukaryotes [26]–[29]. In contrast, one of the most difficult things to explain is the mechanism by which HGT events occur, especially among unrelated species that come from different kingdoms. There is no direct evidence of a mechanism that enables HGT but some hypotheses have been proposed. For example in fungal HGT, vectors such as mycoviruses, plasmids and transposable elements have been proposed to explain this phenomenon [30]. Also, the physical interaction between symbiotic or host-parasite organisms have been suggested as a way for the transfer to occur [31]. Experimental evidence is still needed to confirm or reject these hypotheses.

There are very few reports of HGT from plants to fungi. The *Av1* gene of *Verticillium dahliae* and their homologs in other plant pathogens was presented as potential candidate of HGT from plants to fungi [32]. Strong evidence for four possible events of HGT from plants to fungi were provided by [33]. These proteins were predicted as Zinc binding alcohol dehydrogenase, DUF239 domain protein, Phosphate-responsive 1 family protein and a hypothetical protein with similarity to zinc finger (C2H2-type) protein.

In this study we provide evidence for the presence of a plant-like S8A subtilisin in the genomes of several species of the genus *Colletotrichum*. These proteins show evidence of lateral gene transfer from plants to a *Colletotrichum* ancestor. This is the first time that evidence is provided for the horizontal transfer of a plant subtilisin to pathogenic fungi. The expression analysis shows that at least two subtilisins of maize are down-regulated when the CPLS is induced. In view of the wide variety of processes that plant subtilisins are involved, it is possible that *Colletotrichum* acquired and use plant-like subtilisins to manipulate the host metabolism.

## Results

The aim of this study was to determine whether genes from plants have been horizontally transferred members of the genus *Colletotrichum*. Our first step to identify potential horizontally transferred genes was to perform a battery of BLAST searches against a database composed of all proteomes available in the UniProt database (www.uniprot.org) using the *C. graminicola*, *C. higginsianum* and *C. gloesoporioides* proteins as query sequences. These BLAST searches resulted in the identification of one protein from *C. graminicola* (locus tag, GLRG_05578; GenBank accession number EFQ30434) and two from *C. gloeosporioides* (locus tags CGLO_07890 GenBank accession number KC544259 and CGLO_10271 GenBank accession number KC544258, genome project number SUB133583) with high percentages of BLAST hits in the kingdom Viridiplantae. We performed additional BLAST searches using these three protein sequences versus other databases (see materials and methods) but no evidence of homology to fungal proteins was found. The proteins GLRG_05578 and CGLO_07890 were identified as members of the subtilisin S8A family and they were designated as *Colletotrichum* plant-like subtilisins (CPLSs). The protein CGLO_10271 appears to be a truncated CPLS. By analyzing the DNA sequence of this gene we determined that a premature stop codon truncates the protein’s translation. By aligning the three gene sequences, we identified a thymine at position 1470 downstream of the start codon that caused a frame shift in the open reading frame, which resulted in a premature stop codon. The presence of this premature stop codon was confirmed after PCR amplification and sequencing of the genomic region. We performed TBLASTN searches of the three *Colletotrichum* genomes using the CPLSs as query sequences to identify the presence of possible CPLS pseudogenes, but no evidence was found.

Interestingly, these BLAST searches failed to identify CPLSs in the genome of *C. higginsianum* leading us to speculate that this species lacks a copy of this gene. To confirm that the *C. higginsianum* genome lacks a CPLSs, we performed TBLASTN searches of the *C. higginsianum* RNA-Seq sequence reads [2] and identified sequences homologous to the *C. graminicola* and *C. gloeosporioides* CPLS. These results indicate that like the other *Colletotrichum* spp. that we examined, *C. higginsianum* also contains a CPLS. In addition, a BLASTP search of the predicted protein sequences of *C. acutatum* (genome sequence kindly provided by R. Baroncelli) revealed the presence of an ortholog in this species as well. Also, a TBLASTN search of the assembled genome of *C. sublineolum* (Rech and Thon, unpublished data) showed evidence of a CPLS in this species.

We considered the possibility that the CPLSs may in fact belong to contaminating DNA samples in the genome sequencing projects. To determine whether the CPLSs could have been contamination, we examined the position of GLRG_05578 in the genome assembly of *C. graminicola* and the genes in its vicinity. Gene GLRG_05578 is located on supercontig 1.19 in contig 122 of the *C. graminicola* genome project (BioProject: PRJNA37879). Contig 122 is 192 Kb in length and has 64 predicted genes. A BLAST search of the flanking genes (GLRG_05577 and GLRG_05579) revealed that the most similar sequences in GenBank are from other fungi (Figure S1 in File S1). From this result we conclude that contig 122 is, in fact, from the genome of *C. graminicola*. If GLRG_05578 is from contamination, then the contaminating sequence would have to have been aligned and assembled into the fungal genomic sequences during genome assembly. Since transposable elements (TEs) frequently cause misassemblies, we determined whether there are TEs flanking GLRG_05578. The closest annotated TE is located 7 Kb downstream of the gene (data not shown) and is unlikely to have caused misassembly GLRG_05578. Furthermore, the CPLSs are found in the genome sequence of four additional species of *Colletotrichum*, all of which were sequenced by different research groups at different institutions. It is unlikely that the same contaminating sequence would be encountered in all of the genome sequencing projects.

**Figure 1:**
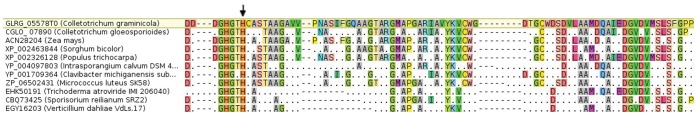
Representative portion of a multiple sequence alignment of CPLSs and subtilisins from plants, bacteria and fungi. The three best BLAST hits to GLRG_05578 from each taxonomic group were used to create the alignment. Amino acid disagreements to GLRG_05578 are represented by dots. Gaps are represented with a dash symbol. The arrow over the alignment indicates the position of the conserved histidine residue of the catalytic site of subtilisins.

The BLAST searches of the CPLSs to the GenBank nr database revealed that the CPLSs are most similar to plant proteins with the most similar plant BLAST hit having 51.2% identity while the most similar bacterial, archeal and fungal hits were 33.3%, 19.8%, and 24.1% identical respectively. The global multiple sequence alignment between the CPLSs and plant subtilisins reveals that the CPLSs have between 40% and 50% identity to their plant counterparts. In general, subtilisins belonging to the same family have conserved residues at the catalytic site, Asp, His, Ser in all organisms. The plant-like subtilisins identified in *Colletotrichum* spp. also show conserved residues at the catalytic sites when compared to their plant counterparts. In contrast, these same regions were less conserved in other subtilisins from bacterial or fungal origin (Figure 1).

### Phylogenetic Analysis

**Figure 2:**
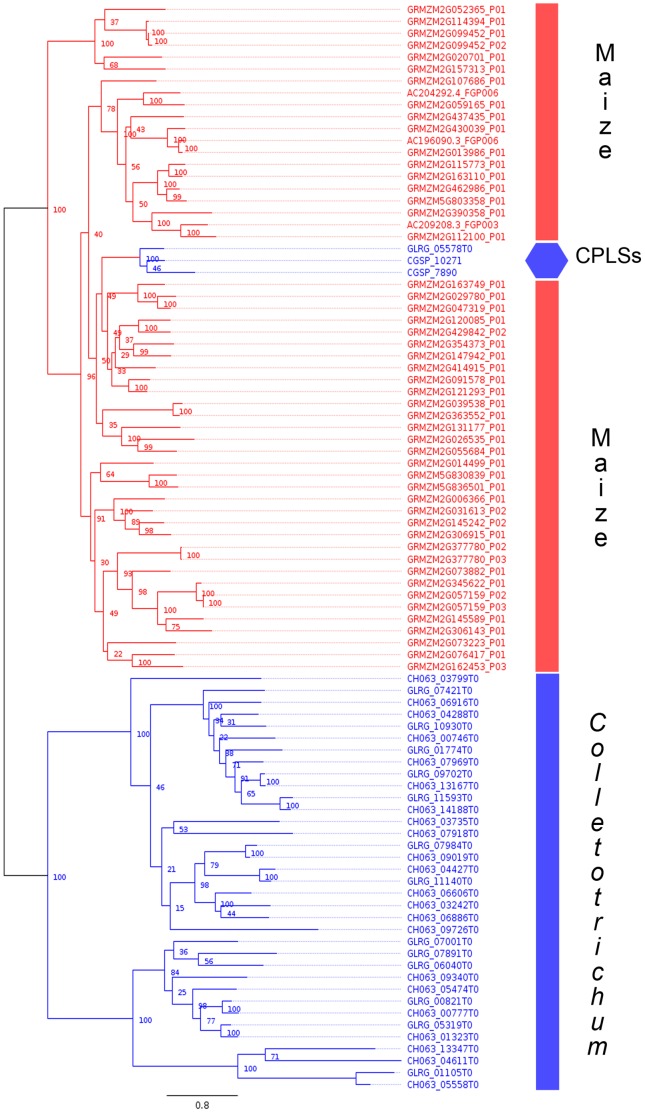
Phylogenetic tree of subtilisins of *Zea mays* (GRMZM2G or AC) colored in red and *Colletotrichum graminicola* (sequence IDs beginning with GLRG) and *C. higginsianum* (CH) colored in blue. Internal nodes are labeled with percentage of bootstrap support.

We constructed phylogenetic trees to test the hypothesis that the CPLSs are derived from plants by HGT. The S8A subtilisins are abundant in all of the kingdoms of life. For that reason we selected a subset of the most similar sequences to our candidates from Bacteria, Archaea, Metazoa, Fungi and Viridiplantae to reconstruct the phylogenetic tree (Figure S2 in File S1). This tree shows that the CPLSs share a common lineage with subtilisins from plants while the remaining *Colletotrichum* subtilisins share a common lineage with other fungal subtilisins. To confirm this, all of the S8A subtilisins from *C. graminicola, C. higginsianum* and *Zea mays* were identified using the MEROPS server (http://merops.sanger.ac.uk/cgi-bin/batch_blast). The S8A protein sequences were aligned with MAFFT [34] and the alignment was manually edited (removing sites with high percentages of gaps) using Geneious 5.5.7 [35]. A maximum likelihood tree was reconstructed with PhyML [36], and the tree was tested by performing a non-parametric bootstrap analysis with 100 replications. The maximun likelihood tree shows the separation of the maize subtilisins and *Colletotrichum* subtilisins into two clades with the CPLSs within the clade of maize subtilisins (Figure 2). To further confirm these results, we tested the tree with several topology tests by constructing a new tree that forced the monophyly of the fungal subtilisins together with the CPLSs. MrBayes [37] was used to reconstruct and constrain the tree. TREE-PUZZLE [38] was used to perform the ELW (Expected Likelihood Weights) topology test and CONSEL [39] was used to perform the AU (Approximately Unbiased) and SH (Shimodaira and Hasegawa) topology tests. In all of the topology tests the unconstrained tree was not rejected and the monophyletic fungal tree was rejected at the 95% confidence level. These results support the hypothesis that the CPLSs share a common ancestry with the plant subtilisins that is distinct from the other subtilisins in the *Colletotrichum* genomes.

Subtilisins from bacteria were the best BLAST hits to the CPLSs after those from plants. To determine the relationship between CPLSs and bacterial subtilisins, a second tree was reconstructed with all S8A subtilisins of *C. graminicola, C. higginsianum* and *Zea mays* plus some representatives of the most similar bacterial subtilisins. The resultant tree shows three well defined clades: maize sequences including CPLSs, *Colletotrichum* sequences and bacterial sequences (Figure S3 in File S1). This tree also shows a close clustering between bacterial subtilisins with maize sequences. *Colletotrichum* subtilisins (excluding plant-like subtilisins) form a well-defined clade but this is more distant to the other two.

**Figure 3:**
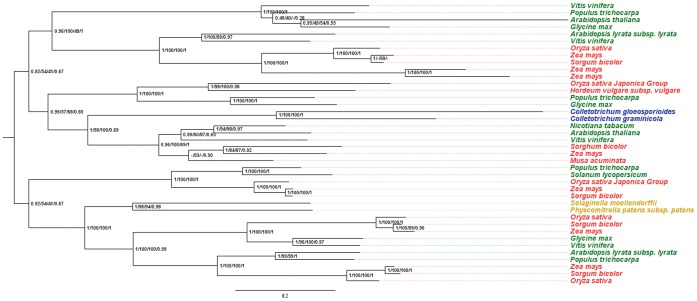
Location of the CPLSs in the phylogenetic tree of plant subtilisins. The tree was rooted on one of the multiple duplication events in this family. The colors represent the taxonomic groups: red for monocots, green for dicots, yellow for embryophytes and blue for *Colletotrichum*). The numbers at each node represent the posterior probability/percentage of bootstrap in PhyML/percentage of bootstrap in RAxML/SH-branch test.

Plant-like subtilisins are absent in all other species of fungi, including *Verticillium* spp. *Verticillium* is estimated to have diverged from *Colletotrichum* approximately 150 million years ago [2]. We constructed a plant subtilisin phylogeny that included the CPLSs, to better understand when, during the evolution of plants, the CPLSs where likely to have been transferred to *Colletotrichum* and to determine if this date occurred after the divergence of *Verticillium* and *Colletotrichum*. The plant S8A subtilisins show evidence for several duplication events but with a considerable level of conservation. We selected the most similar plant subtilisin sequences to CPLSs available in GenBank [40] to construct a tree that shows the position of the CPLSs in the plant subtilisins group. The sequences were aligned with MAFFT and edited manually (deleting highly divergent domains). We also prepared alignments by editing the MAFFT alignment with trimAl [41] and Gblocks [42] and constructed four phylogentic trees using PhyML (Figure S4 in File S1).The tree constructed using the manually edited alignment was the only tree that had the same topology as the tree constructed form the unedited alignment. In addition, the bootstrap support values in the tree constructed from the manually edited alignment were higher than the values from the unedited alignment. Therefore, we selected the manually edited alignment for further analysis. Next, we constructed phylogenetic trees using several different methods. A Bayesian tree, supported by posterior probability index, was constructed using MrBayes [37]. A maximum likelihood tree supported with a fast bootstrap approximation was constructed using RAxML [43]. A maximum likelihood tree was constructed using PhyML [36] with full non-parametric bootstrap and SH-branch tests [44] to verify the position of branches inside the tree (Figure 3). *Colletotrichum* plant-like subtilisins were placed in the same position of the tree in all methods tested. In these trees, the CPLSs are in a position that is ancestral to a lineage that gives rise to monocot and dicot lineages, suggesting that the CPLS were transferred to *Colletotrichum* some time before the divergence of monocots from the angiosperms approx. 134 million years ago (Myr) [45] to 200 Myr [46] with the most recent estimates of 155 Myr to 145 Myr [47]–[49]. According to these dates, the HGT event would have occurred approximately 150 to 155 Myr, just before the monocot divergence and just after the *Verticillium-Colletotrichum* divergence.

### Domain Content of CPLSs

**Figure 4:**
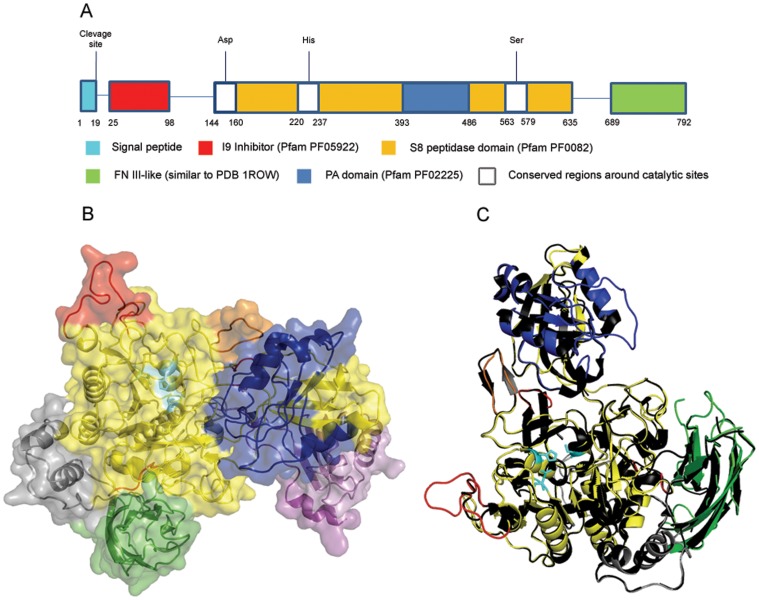
a) Schematic view of protein domains found in *C. graminicola* subtilisin GLRG_05578. b) 3d surface view of the protein GLRG_05578. The peptidase s8 domain is colored in yellow, PA domain in blue and Fn III-like domain in green. The ß-hairpin like domain is colored in orange, the residues of the catalytic site are colored in cyan and the putative sites of Ca^+^ replacement are in red. The signal peptide and I9 inhibitor are in pink and violet respectively. The gray residues have not been assigned any domain c) Alignment between mature forms of subtilisin SBT3 of tomato and GLRG_05578 of *C. graminicola*. The tomato subtilisin is in black and the *C. graminicola* subtilisin is colored as in (b).

All of the plant S8A subtilisins that we analyzed contain three domains, the inhibitor I9 domain (PF05922), the PA domain (PF02225) and the peptidase S8 domain (PF00082). The same three domains were observed in CPLSs (Figure 4a). Other domains, such as DUFF1034 (PF06280) and Pex16 (PF08610) are present in some plant subtilisins, but are absent in CPLSs. The peptidase S8 domain is always present in fungal subtilisins and is accompanied by either domain PA or inhibitor I9 but rarely with PA and I9 at the same time. Thus, the domain arrangement in CPLSs is more similar to subtilisins from plants and bacteria than to their fungal counterparts. Additionally, in CPLSs a signal peptide and a cleavage site were predicted by WoLF PSORT [50] and SignalP [51]. This finding suggests that, like many subtilisins, the proteins are secreted.

Using the classification system of PANTHER [52], we determined that all proteins related to CPLSs are included in the same sub-family (PTHR10795:SF17), and no other fungal protein was included into this sub-family. Only CPLSs and subtilisins from plants and bacteria are assigned to PTHR10795:SF17. With all the sequences identified as members of PTHR10795:SF17 a profile hidden Markov model (HMM) was constructed with HMMER [53]. The profile HMM was used to search the nr database with the tool hmmsearch of the HMMER web server (http://hmmer.janelia.org/search/hmmsearch). This search only resulted in hits from plants, followed by the bacterial phyla Actinobacteria, Gammaproteobacteria and Chloroflexi. These results demonstrate the resemblance of CPLSs to plant proteins.

### CPLS Structure Modeling

We hypothesized that the CPLSs might still share common structural features with their plant counterparts. Common structure may be used to imply common function [54]. Recently, the plant subtilisin SBT3 (PDB 3I6S) from tomato was crystallized [55] and this was used to predict the structure of different S8A subtilisins of Arabidopsis and the pathogenesis related protein P69B of tomato [56]. Protein SBT3 shares 46,7% identical sites with the GLRG_05578 protein of *C. graminicola*. We reconstructed the tertiary structure of GLRG_05578 (Figure 4b) using the Phyre 2 server [57] and then used the resulting structure to perform a search using the Dali server [58]. The Dali server returned a match to the tomato SBT3 structure with a root-mean-square deviation (RMSD) of 0.7 Å and a Z-score of 65.9 indicating that the structures are highly similar. We also aligned the two structures using PhyMol [59] resulting in a series of RMSD values ranging from a maximum RMSD of 11.68, to a minimum of 0.02 (Figure S5 in File S1). The most relevant regions and sites of SBT3 protein described in [55], [56], [60] were also observed in GLRG_05578 (Table 2, figure 4c). One of those is the beta hairpin, described in [55] as an essential structure for the homo-dimerization of SBT3. The GLRG_05578 structure is similar, but with the beta sheet folding not well defined. Three defined regions Ca-1 (Gly-225-Gly-243), Ca-2 (Lys-498) and Ca-3 (Cys-170-Cys-181) were reported as potential responsible sites for the stabilization of SBT3 to high temperatures and alkalinity (only the relevant residues for each site were named in parenthesis). These sites could act in replacement of Ca^2+^, which is the element commonly present in subtilisins to perform the stabilization [55]. The lysine 498 in SBT3 is conserved in GLRG_05578 (Lys 527), sharing the same relative position in the peptidase S8 domain (Figure S6 in File S1). Regions Ca-1 and Ca-3 of SBT3 present alignment similarities with GLRG_05578 (RMSD 0.311 for Ca-1 region and RMSD 0.258 for Ca-3 region). But minor shape differences could be observed (Figure S7a and S7b in File S1). The catalytic triads (Asp 144/His 215/Ser 538 in SBT3 and Asp 149/His 227/Ser 566 in GLRG_05578) are placed in the same position in both structures. Finally, one of the residues apparently responsible for the union of PA domains in the dimerization of SBT3 (Arg 418) is not present in *C. graminicola* subtilisin. However, GLRG_05578 has some of the elements for the interaction with another monomer, like the hairpin and the PA domain (structures observed in the SBT3 dimer conformation).

**Table 2:** Comparison of domains and sites between SBT3 (PDB 3I6S) and the predicted tertiary structure of GLRG_05578.

Region	SBT3		GLRG_05578	
	start	end	start	end
Domain peptidase S8	113	599	141	603
PA domain	363	457	393	486
Beta hairpin (like)	519	528	547	558
Domain Fn III-like	600	761	689	792
Hypothetical site of calciumstability (Ca-1)*	225	243	237	254
Hypothetical site of calciumstability (Ca-3)*	170	181	175	186
**Relevant sites**	**Residue**		**Residue**	
Catalytic site	Asp 144		Asp 149	
Catalytic site	His 215		His 227	
Catalytic site	Ser 538		Ser 566	
Hypothetical site of calciumstability (Ca-2)*	Lys 498		Lys 527	

* Only relevant residues were named.

Another 3D structure of a *C. graminicola* subtilisin was reconstructed. Protein GLRG_07421 is the most similar subtilisin to GLRG_05578 in the *C. graminicola* proteome with 24% amino acid identity. The differences between these proteins are evident in the 3D alignment (Figure S8 in File S1). Only the surrounding regions of the catalytic sites show resemblances. These results show the uniqueness of GLRG_05578 in the *C. graminicola* subtilisin arsenal.

### CPLS Gene Expression

**Figure 5:**
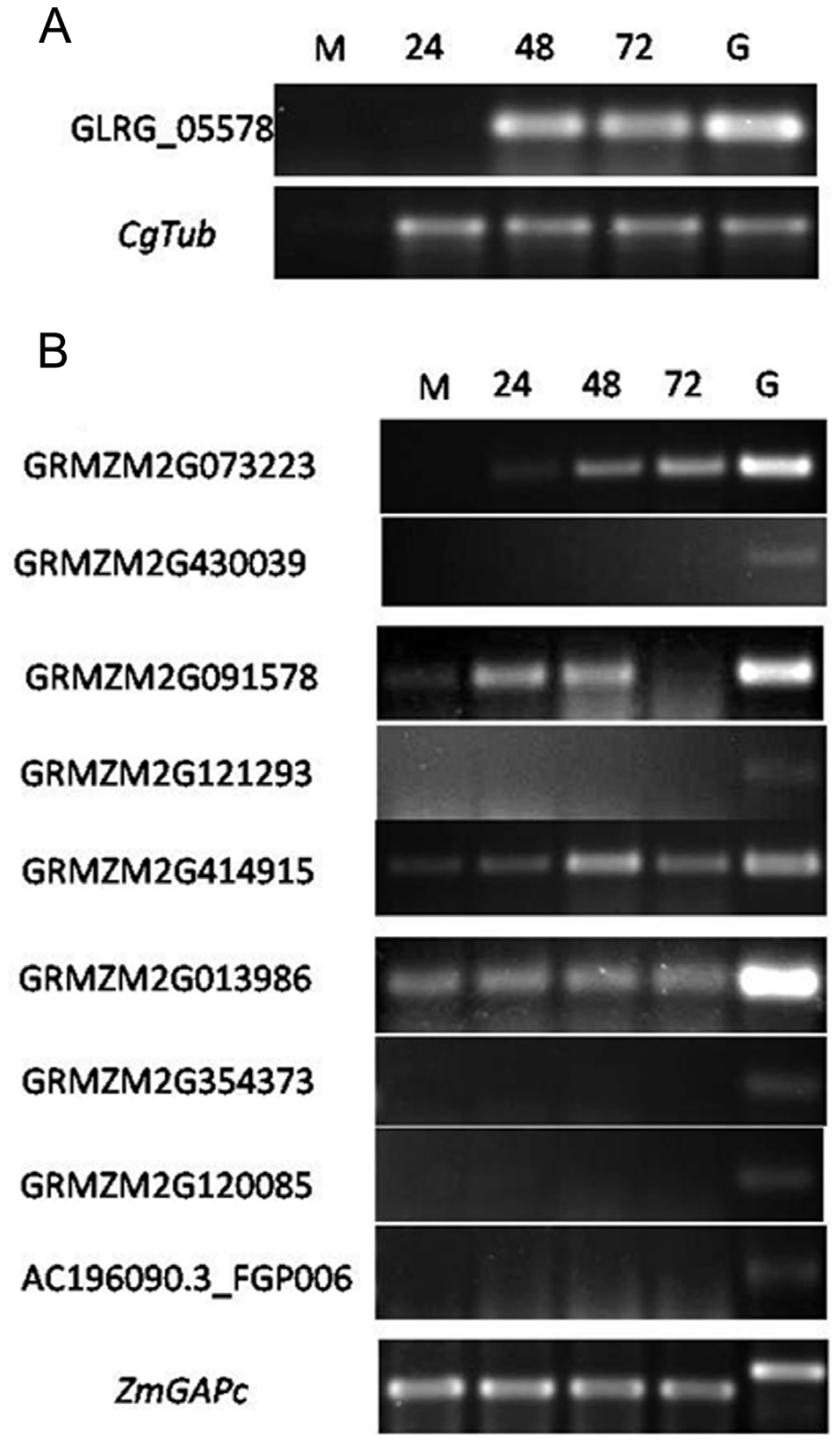
Gene expression during anthracnose development. Due to the low representation of fungal mRNA in the samples, semi-quantitative RT-PCR assays were conducted to test the expression of CPLS GLRG_05578 of *C. graminicola* and the selected maize putative subtilisins. The amount of total RNA used in each PCR reaction was adjusted to the amount needed to provide equal amplification levels of CgTub in all samples. PCR products were visualized after electrophoresis on 2% agarose gel and ethidium bromide staining. **a**) RT-PCR products for GLRG_05578 and CgTub. **b**), RT-PCR products of nine genes encoding putative subtilisins in maize. *ZmGAPc* was amplified as an internal loading control. The number of cycles in PCR reactions was optimized to be in the linear amplification range of each gene. These assays were repeated two times with similar results. In both panels, the numbers over the lanes indicate the time-point at which RNA samples were taken. M indicates RNA samples from mock-inoculated leaves and G indicates genomic DNA.

Subtilisin is a large family in plants, and in the case of maize we identified 53 members of the subtilisin S8 family. A recent study described the expression pattern of a set of subtilisin S8A genes, also called PR-7, in maize leaves infected with *Ustilago maydis* [61]. To further investigate the participation of subtilisin-encoding genes during anthracnose development, we followed the expression of ten putative PR-7 genes in the maize genome as well as GLRG_05578 during infection by *C. graminicola*. The selection of the ten genes was based on the identity with CPLSs, the identity with P69s (a well-known group of PR-7 genes) in tomato, [12], [15] and the expression pattern after *U. maydis* infection. The expression assays revealed that the CPLS GLRG_05578 of *C. graminicola* is induced at late stages of biotrophic infection (48 hours post-infection) and continues to be up-regulated 72 hours post-infection (hpi) (Figure 5a), suggesting the importance of the protein product during the transition from biotrophic to necrotrophic stages of the fungal infection.

In the case of the maize genes tested a heterogenic behavior was detected. For instance no amplification product was detected for five of the sequences (GRMZM2G430039, GRMZM2G121293, GRMZM2G354373, GRMZM2G120085, AC196090.3_FGP006) suggesting that the protein product of these genes is not needed during infection by *C. graminicola* (Figure 5b). In contrast, GRMZM2G013986 displays constitutive expression with no changes between mock-inoculated leaves and leaves from plants infected with *C. graminicola*.

Gene GRMZM2G073223 displays a similar expression pattern as other maize PR genes such as PR-1, PR-4 and PR-5 during anthracnose development [62] (Figure 5b).

The expression results also revealed interesting expression profiles for two maize genes GRMZM2G091578 and GRMZM2G414915, which display increased levels of expression at early stages of infection that decreased with the progress of the disease (Figure 5b). Among the putative subtilisin-encoding genes from maize GRMZM2G091578 and GRMZM2G414915 are among the most similar to CPLS GLRG_05578 of *C. graminicola*. The fact that these maize genes are down-regulated at the time the fungal homolog is induced might suggest a compensation of the enzymatic activity where the fungus is hijacking the plant subtilisins, interfering with the normal proteolytic activities in the host cells and the biochemical processes associated with specific forms of subtilisins.

While the RT-PCR experiments were designed to study the expression of the CPLS in *C. graminicola*, they also provide additional evidence that GLRG_05578 is not a product of foreign DNA contamination in the *C. graminicola* genome project. We detected the expression of GLRG_05578 in infected maize leaves (Figure 5a). A Primer-BLAST search (http://www.ncbi.nlm.nih.gov/tools/primer-blast/) failed to detect any potential primer binding sites in the maize genome. In addition, we amplified the gene fragment from genomic DNA obtained from axenic culture of *C. graminicola* (Figure 5a).

## Discussion

Phylogenetic analysis, domain content and tertiary structure prediction allowed us to identify the presence of a plant-like member of the subtilisin S8A family in the genome of *Colletotrichum graminicola* and *Colletotrichum gloeosporioides*. We have also evidence of CPLSs in *C. higginsianum*, *C. acutatum* and *C. sublineolum*. Several independent lines of evidence support the hypothesis that CPLSs are part of *Colletotrichum* genomes and not a product of contamination from plants or other foreign DNA. Most importantly, the presence of CPLSs in the genomes of five difference species of *Colletotrichum*, all of which were sequenced by different research groups in different laboratories using different samples and methodologies support the hypothesis that the CPLSs are not a result of contamination and are, in fact, components of the *Colletotrichum* spp. genomes.

The most similar sequences to the CPLSs are found in plants followed by bacteria. The phylogenetic reconstruction shows that CPLSs are within a clade of plant subtilisins. This suggests that the CPLSs originated in plants, and were not transferred vertically from fungi. On the other hand, bacterial S8A subtilisins (mainly from Actinobacteria, Cloroflexi and Gammaproteobacteria) were observed in BLAST searches as the most similar proteins after plants. Phylogenetic reconstructions place a monophyletic bacterial branch near to the plant-CPLS lineage (Figures S2 and S3 in File S1). This reveals a complex evolutionary history behind these proteins. In fact, CPLSs, plant subtilisins and some bacterial subtilisins (of the three phyla named earlier) are recognized as members of PTHR10795:SF17 subfamily in the PANTHER database [52]. Most of the bacterial proteins identified as S8A subtilisins in MEROPS belong to the PTHR10795 family in PANTHER but only a few belong to the PTHR10795:SF17 subfamily. The classification system in PANTHER uses experimental data and evolutionary relationships to create families. Functional divergence evidence from the ancestors is used to classify proteins in subfamilies [52]. In MEROPS, the proteins are classified on the basis of sequence comparison (BLAST, FastA, HMMER) to a reference sequence [9]. Subfamily S8A is equivalent to PTHR10795, but PTHR10795 sub-family 17 has no equivalent in the MEROPS classification system.

Members of subfamily PTHR10795:SF17 only could be identified in three phyla of bacteria. A systematic loss of subfamily PTHR10795:SF17 in bacteria and a cross-kingdom HGT event could explain these observations. On the other hand, complex events of HGT from plants to, at least three different, bacterial ancestors could be an alternative but less plausible explanation. In any case, the relationship between bacterial and plants subtilisins cannot be determined with the evidence provided in this investigation. However, other examples of HGT that involve three different kingdoms have been reported in the past [63]. Therefore, we do not discard the hypothesis that subtilisins were transferred horizontally from bacteria to plants, and subsequently to fungi. The lateral transfer of an ancestral subtilisin from bacteria to plants would have happened early in the evolution of plants. Subtilisins from sub-family PTHR10795:SF17 were found in all Viridiplanteae members except in Chlorophyta. This observation reflects the ancient origin of this subfamily in plants.

Likewise, the lateral transfer of a plant subtilisin to a *Colletotrichum* ancestor should be ancient, at least before the divergence between monocots and dicots. Based on the draft genome sequences of members of *Colletotrichum* available to us at this time, CPLSs homologs were found in all five species that we examined. If our hypothesis about the HGT to an ancestor of the *Colletotrichum* is correct, then we expect that all members of the genus to contain a CPLS. If the HGT event occurred earlier, then we expect to find CPLS homologs in other fungal genera. To explain the presence of CPLSs only in *Colletotrichum* without HGT requires us to accept that it is a very ancient gene family that was conserved only in *Colletotrichum* and was lost in all other fungal lineages. We believe that this is unlikely and that the body of evidence supports the HGT hypothesis. The period of time proposed for the HGT transfer is congruent with molecular clock estimation for the monocot divergence from the angiosperms and *Colletotrichum* divergence from other related genera. The estimated divergence date for the monocots ranges from 200 Myr [46] to 134 Myr [45]. The most recent calculations propose intermediate values between 155 Myr to 145 Myr [47]–[49]. For *Colletotrichum* genera the divergence from other members of the class Sordaryomycetes is calculated to be approximately 150 Myr ago [2]. The hypothesis of a lateral transference from an angiosperm that predates the monocot divergence to a *Colletotrichum* ancestor explains the lack of homologous sequence of CPLSs in other fungal species and the abundance in plants. Multiple duplication events prior the monocot divergence are evident in the plant subtilisins tree (Figure 3), but CPLSs are not placed inside any branch of a specific group. The CPLSs are placed at a node ancestral to the monocot divergence. These kinds of ancient HGT events were reported in the past [32], [63]–[66]. Despite the age of the HGT event, the level of conservation of protein sequences is remarkable. This fact also denotes the possibility of protein function conservation in the fungi, probably favored by selection. In fact, expansions of S8A serine proteases have been observed in *C. higginsianum* [2], suggesting an important selection pressure for new copies of this gene in some *Colletotrichum* spp.

The broad spectra of metabolic processes that subtilisins are involved in make it difficult to predict a specific function of the CPLSs. However, the remarkable level of structural conservation of CPLSs with plant subtilisins and the differences with the rest of *Colletotrichum* subtilisins suggests the possibility of molecular mimicry. For parasitic organisms a mimetic molecule is defined as a factor that resembles the host molecules for the pathogen’s advantage [67]. In bacteria, some cases of plant protein mimicry have been reported. AvrPtoB is a protein from *Pseudomonas syringae* than mimics E3 ubiquitin ligase of its plant host [68]. The molecule suppresses programmed cell death in compatible interactions, enabling the pathogen to avoid the hypersensitive reaction. AvrPtoB can also suppress program cell death in yeast, demonstrating that the molecule has different functions in different eukaryotic models [69]. But, at the moment, no cases of fungal proteins that mimic plant proteins have been reported. We are actively investigating the function of CPLSs in *Colletotrichum*, in order to address the mimicry hypothesis.

Our analysis reveals that GLRG_05578 is up-regulated during the infection of maize at 48 hpi and 72 hpi. At the same time, the putative PR-7 genes of maize GRMZM2G091578 and GRMZM2G414915 were induced in the first hours post-infection and then repressed when GLRG_05578 was induced. Putative proteins of GRMZM2G091578 and GRMZM2G414915 genes are S8A subtilisins within the subfamily PTHR10795:SF17 and show the highest similarity to GLRG_05578. In equivalent expression experiments only a few predicted PR-7 proteins were induced or repressed in maize after *Ustilago maydis* infection [61]. In these experiments GRMZM2G091578 expression was detected 4–8 days post-infection (dpi) and GRMZM2G414915 was repressed 4 dpi. This observation shows a differential behavior of maize subtilisins in the presence of two different fungal pathogens. The low and delayed expression of PR-7 in maize in the presence of *U. maydis* is consistent with the behavior of the maize-*U. maydis* pathosystem, because *U. maydis* is an obligate biotroph with longer periods of symptomless colonization compared with *C. graminicola* (a hemibiotrophic fungus). On the other hand, GLRG_05578 was expressed towards the transition from biotrophic to necrotrophic stages of fungal infection and coincide with the down-regulation of two putative maize PR-7s with high sequence similarity to CPLSs. Whether the maize proteins are down-regulated by the effect of GLRG_05578 or by the effect of any other stimulus is not yet known. But the synchrony of induction-repression patterns and the level of similarity suggest an important role of GLRG_05578 in the infection process, perhaps by the repression of pathogen-related proteins in the host. In consequence, the acquisition of a PR-like protein would be important for the fungal cells to interfere with plant immune systems.

The structural similarity of CPLSs with plant subtilisins and the pattern of expression in plant infections suggest an important function in plant-fungal interactions. The direct interaction of proteins has been shown in plant and fungal chorismate mutases of maize and *Ustilago maydis* [70]. The interaction between CPLSs and plant S8A subtilisins is also possible. For example the crystallized subtilisin SBT3 of tomato reveals the conformation of homodimers [55]. Some of the regions involved in the dimerization of SBT3 are present in GLRG_05578 (see Table 2). Whether the CPLSs form dimers is not yet known, but if they do, it is possible that they form heterodimers with their plant counterparts. On the other hand, pre-processed subtilisins are known to inhibit the activity of mature subtilisins. This was studied in a heterologous system, in which the authors determined that the immature form of ARA12 (a subtilisin of Arabidopsis) can inhibit the activity of cucumisin, a subtilase of *Cucumis melo* [71]. The CPLSs encode a signal peptide and the pre-domain inhibitor I9. These domains of the immature proteins are normally removed in the endoplasmic reticulum by auto-catalysis, according to the behavior observed in several studies [72]–[75]. To use the inhibitory property of immature subtilisins observed in ARA12, would require that *Colletotrichum* skips the preprocessing of CPLS that is normally observed in other serine proteases. Experiments are planned to determine whether this is the case.

The case of the *C. gloeosporioides* gene CGLO_10271 is particularly interesting. An insertion of one nucleotide truncates this gene by causing a shift of the reading frame. The DNA sequence is very similar to CGLO_07890 and GLRG_05578 (60.5% and 63%, respectively). Thus, apart from the shift of the reading frame no other nonsense mutations could be identified before or after the nucleotide insertion. Also if the reading frame is corrected, the resulting translated protein shows a high percentage of identity with CGLO_07890 and GLRG_05578 (57.1% and 65.4% respectively). These data suggest that the frameshift mutation is recent. Probably the presence of a second copy of a CPLS is not essential in the genome of *C. gloeosporioides* or the nonsense mutation was a casual event.

Only a few examples of HGT from plants to fungi have been described to date demonstrating that HGT events are very rare [32], [33]. In the case of the CPLSs, the functions of known subtilisins coupled with the expression pattern during plant infection suggest that they have important roles in plant disease. It is interesting to speculate that the role in plant disease provided a selective advantage to the *Colletotrichum* ancestor providing it with improved fitness, possibly with improved ability to invade its host.

## Materials and Methods

### Identification of HGT Events

A BLASTp [76] search was done (e-value threshold: 10^−5^) using the predicted protein sequences from the *Colletotrichum graminicola*, *Colletotrichum higginsianum* [77] and *Colletotrichum gloeosporioides* proteomes (the *C. gloeoposioides* predicted protein sequences were kindly provided by N. Alkan and D. Prusky). Annotated proteins from organisms with complete proteomes deposited in UniProt (www.uniprot.org) were used as the initial BLAST database. The proteins with 80% or more of the BLAST hits from members of the Viridiplantae were selected as candidates. After the first round of candidates was identified, other databases were used to verify the absence of putative homologues not detected in the UniProt database. The NR and EST databases from NCBI (www.ncbi.nlm.nih.gov) and all fungal proteomes of the Broad Institute (www.broadinstitute.org) and the Joint Genome Institute (www.jgi.doe.gov) were used in this second round of searches. The results were analyzed automatically with Python scripts, taking in account the taxonomy of BLAST hits and comparing these with the taxonomy of *Colletotrichum* genera. The percentage of hits with a Viridiplantae taxonomy label was reported.

### Phylogenetic Analysis

The protein sequences used for the phylogenetic reconstruction came from nr database of NCBI (www.ncbi.nlm.nih.gov), Join Genome Institute (www.jgi.doe.gov) and MaizeSequence (www.maizesequence.org/index.html). Using the candidates as a query, BLAST hits (e-value threshold: 10^−10^) with at least 30% of identity and 70% of coverage were used for further analyses. From these, only family members of subtilisins S8A according to MEROPS [9] were chosen. The sequences were submitted to the PANTHER classification system [52] to recover only the members of PANTHER’s family PTHR10795 sub family 17. The selected sequences were aligned with MAFFT v6.814b [34] and then manually edited to remove highly divergent alignment columns. Two alternative alignments were also prepared using Gblocks [42] and trimAl [41] (Figure S4 in File S1). The percentage of unresolved quartets was used as measure of the contribution of each sequence to resolve the topology of the phylogenetic tree. Using the program TREE-PUZZLE [38] the alignment was analyzed to ensure that all sequences had less than 10% unresolved quartets. Any sequences with more than 10% of unresolved quartets were removed of the analysis. MODELGENERATOR [78] was used to predict an accurate model of sequence evolution and matrix of substitution from the dataset. A maximum posterior tree was constructed with MrBayes [37], performing 2,000,000 generations of samples, using the substitution matrix and model predicted by MODELGENERATOR but allowing the program to calculate the proportion of invariable sites and the alpha parameter for gamma distribution. Two Multiple Chain Markov Chain Monte Carlo (MCMCMC) searches were conducted with four chains each (three heated and one cold). The convergence between them was checked using a sample frequency of 1000 generations. A burn-in of 25% of generations was excluded to reconstruct the Bayesian consensus tree.

PhyML [36] was used to reconstruct the maximum likelihood tree and perform 100 non-parametric bootstrap replicates and SH-like branch test support. RAxML [43] was used to conduct a rapid Bootstrap analysis with 1000 replicates. Substitution matrix and model selection of MODELGENERATOR were used.

To verify the accuracy of the trees reconstructed, we used statistical topologies test to corroborate the position of the fungal subtilisins inside the plant branches. MrBayes was used to constrain specific groups and generate trees to evaluate different topologies. Expected Likelihood Weight (ELW) test was conducted in TREE-PUZZLE. The AU (Approximately Unbiased) and SH (Shimodaira and Hasegawa) tests were conducted in CONSEL software [39].

To reconstruct the tree of maize and *Colletotrichum* subtilisins, all the sequences identified as subtilisins S8A in MEROPS (http://merops.sanger.ac.uk/cgi-bin/blast/submitblast/merops/advanced) in the proteomes of *Zea mays*, *Colletotrichum graminicola* and *Colletotrichum higginsianum* were used. The same procedure explained earlier was applied with two differences. Only PhyML bootstrap analysis was used to support the topology and the percentage of quartets was not calculated.

### Amplification and Sequencing of Colletotrichum Gloeosporioides CGLO_^10271^ Gene

To verify the presence of a premature stop codon in the putative CPLS CGLO_10271 in the *Colletotrichum gloeosporioides* genome, PCR amplification and sequencing were used. The PCR was performed with PCR extender system Taq polymerase (5 Prime) with forward: AAGCTGCGACGGGGTCAACG and reverse: GCGGCGTCGTCAAGTCTGCT primers for 30 cycles. PCR products were visualized after electrophoresis on agarose gels stained with ethidium bromide. The material for sequencing was isolated from agarose gels, purified, and then sequenced by the Genomics and Proteomics Sequencing Service of the University of Salamanca, using the same primers mentioned above.

### Domain Determination

The PA domain, I9 inhibitor and peptidase S8 domain were identified by Pfam [79]. Fn-III like domain was predicted by visual structural homology with SSP-19 (sperm-specific protein, PDB entry 1ROW). Signal peptide and cleavage site were predicted by WoLF PSORT [50] and SignalP [51]. PANTHER [52] was used to classify the proteins in more specific categories. Profile hidden Markov models were constructed using HMMER [53].

### 3d Structure Determination

The prediction of 3d structures was made in Phyre 2 [57]. The manipulation, structural alignment and comparison between 3d models were done with PhyMOL [59]. Dali pairwise comparison [80] was also used to evaluate the general statistics of the structural alignment. The similarity between the structural regions was evaluated manually and with PhyMOL ColorByRMSD script.

### Gene Expression Assays

Total RNA samples were prepared from maize leaves infected with *C. graminicola* strain M.1001 following the methodology previously described by [62]. Briefly, ten droplets (7.5 ml) containing 3×10^5^ spores/ml were inoculated on the adaxial side (away from the midvein) of the third leaf of maize plants (highly susceptible inbreed line Mo940) in the V3 developmental stage. Plant leaves were harvested 24, 48 and 72 hours post-infection (hpi) and total RNA was prepared using TRIZOL^®^ reagent (Gibco-BRL) according to the protocol provided by the manufacturer.

To assay the gene expression pattern of a set subtilisin S8A genes from maize and *C. graminicola*, semiquantitative RT-PCR experiments were conducted by reverse transcription of RNA followed of PCR reactions using specific primers for each gene. Due to the high sequence identity among the various subtilisin homologs in maize, the specific primers were designed using the predicted 5′UTR region of each sequence. cDNA synthesis was performed using 5 mg of total RNA, Moloney Murine Leukaemia Virus-Reverse Transcriptase (MMLV-RT®, Promega) and oligo-dT primers. Previous to the reverse transcription, RNA samples were treated with Turbo DNA-Free DNAse (Ambion, Austin Texas) to remove trace amounts of genomic DNA.

The amplification of the constitutively expressed beta-tubulin and GAPc genes from *C. graminicola* and maize, respectively, were used as loading and RT controls. PCR reactions were performed in the linear range of product amplification that is between 25 and 35 cycles depending on the abundance of the different target in the samples. To confirm the absence of genomic DNA contaminations RT-PCR assays were performed in reactions where the reverse transcriptase was omitted. PCR products were visualized after electrophoresis on 2% agarose gels and staining with ethidium bromide. Primers used for the PCR reactions are listed in Table 3.

**Table 3:** Primers used for gene expression assays.

Gene	Primer	Sequence	Product Size (bp)
GRMZM2G073223_P01	223 Fw	ATTCCGGTCAGTGCGCAGGC	
	223 Rv	TGCCGTCGTGAACAGCCGTC	421
GRMZM2G099452_P02	452 Fw	GCCAGCACCAGCGGAACTGT	
	452 Rv	CAGTGGGCACCGAGGGAGGA	245
GRMZM2G013986_P01	986 Fw	CCAGCTCACCGCCAGTGCTC	
	986 Rv	TAAGCAGCCGCCTTGGCGTT	210
GRMZM2G091578_P01	578 Fw	GCAGTCACGCCTTCCCGTCC	
	578 Rv	TGGCGCCGCATTGTGAGTGA	204
GRMZM2G354373_P01	373 Fw	TTTTCCCGATCCGGCACCGC	
	373 Rv	GGAGGACAGCAGGCCCAGGA	428
GRMZM2G120085_P01	085 Fw	CCGTCTGTGCACCGGACACC	
	085 Rv	CGCCTCCAGATTGCCCGTGG	273
GRMZM2G414915_P01	915 Fw	GCCGCTGTGCCTAGCTCTCG	
	915 Rv	CTCGGCCTCGTCCTCGTCCA	265
AC196090.3_FGP006	090 Fw	CTCCTCCTGCTGCTGTCCGC	
	090 Rv	TCAGTGAGGCTGGCGGCGAA	230
GRMZM2G121293_P01	293 Fw	GCACGAACATGCGTAACATCGGC	
	293 Rv	GAGCATCTTGGCGGCGGAGG	203
GRMZM2G430039_P01	ZmP69Fw	CGGCGACCGCCTAGCATCTG	
	ZmP69Rv	CGGCGACCGCCTAGCATCTG	212
ZmGAPc	GAPc-F	GCTAGCTGCACCACAAACTGC	
	GAPc-R	TAGCCCCACTCGTTGTCGTAC	500
GLRG_05578	CgSLFw	GCCGATCCCTCATCGCTGCC	
	CgSLRv	GCCGAGCAGGGCCGAGTTTT	230
GLRG_01057 (CgTub)	CtubF	CAGTCCCTTGGGCGGCACAG	
	CtubR	TCCCGGGGCAATTGAACGCC	350

## Supporting Information

File S1 Figures S1–S8.
(DOCX)
