# Supplementary material for: Horizontal Transfer of a Subtilisin Gene from Plants into an Ancestor of the Plant Pathogenic Fungal Genus Colletotrichum
Source: PLoS One. 2013 Mar 15;8(3):e59078. doi: 10.1371/journal.pone.0059078 (PMC3598655; doi:10.1371/journal.pone.0059078)
Supplement: File S1 — Figures S1–S8. (DOCX) [file pone.0059078.s001.docx]

**A**


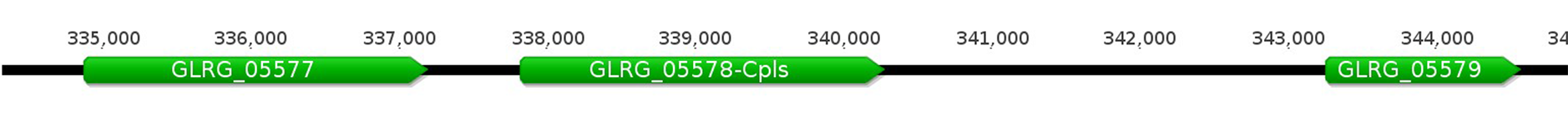


**B**

| acc. number | e-value | Species name | Kingdom |
| --- | --- | --- | --- |
| GLRG_05577 | | | |
| EKV09092 | 0 | *Penicillium digitatum* Pd1 | Fungi |
| XP_002378970 | 0 | *Aspergillus flavus* NRRL3357 | Fungi |
| XP_003043056 | 0 | *Nectria haematococca* mpVI 77-13-4 | Fungi |
| GLRG_05578 | | | |
| XP_003608462 | 0 | *Medicago truncatula* | Viridiplantae |
| XP_002865008 | 0 | *Arabidopsis lyrata* subsp. lyrata | Viridiplantae |
| XP_003550022 | 0 | *Glycine max* | Viridiplantae |
| GLRG_05579 | | | |
| EIN04521 | 3,37E-52 | *Punctularia strigosozonata* HHB-11173 SS5 | Fungi |
| CCA72850 | 2,08E-51 | *Piriformospora indica* DSM 11827 | Fungi |
| XP_001838268 | 6,83E-51 | *Coprinopsis cinerea* okayama7#130 | Fungi |

**Figure S1**. **A)** Diagram of contig 122 from the *Collotetrichum graminicola* M 1001 sequencing project showing the genes flanking CPLS GLRG_05578. **B)** This table shows the best hits from a BLASTp search of the GenBank nr database performed with the three genes shown in A as a query.


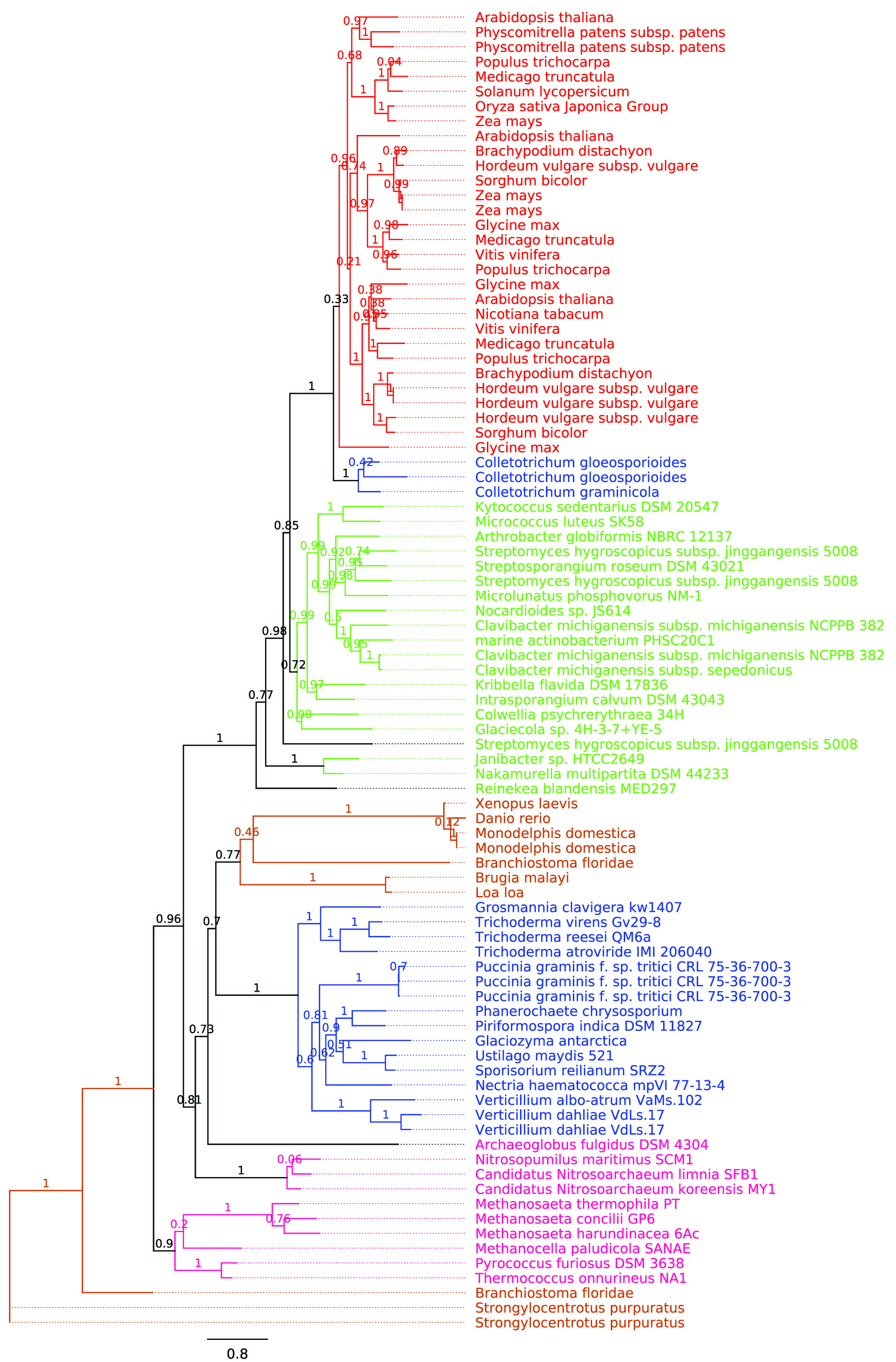


**Figure S2**. Phylogenetic tree of the protein sequences most similar to GLRG_05578 (of *C. graminicola)* from various kingdoms. Fungi are colored in blue, Viridiplantae in red, Bacteria in green, Archaea in pink and Metazoa in light brown. Internal nodes are labeled with SH-like support values.

**
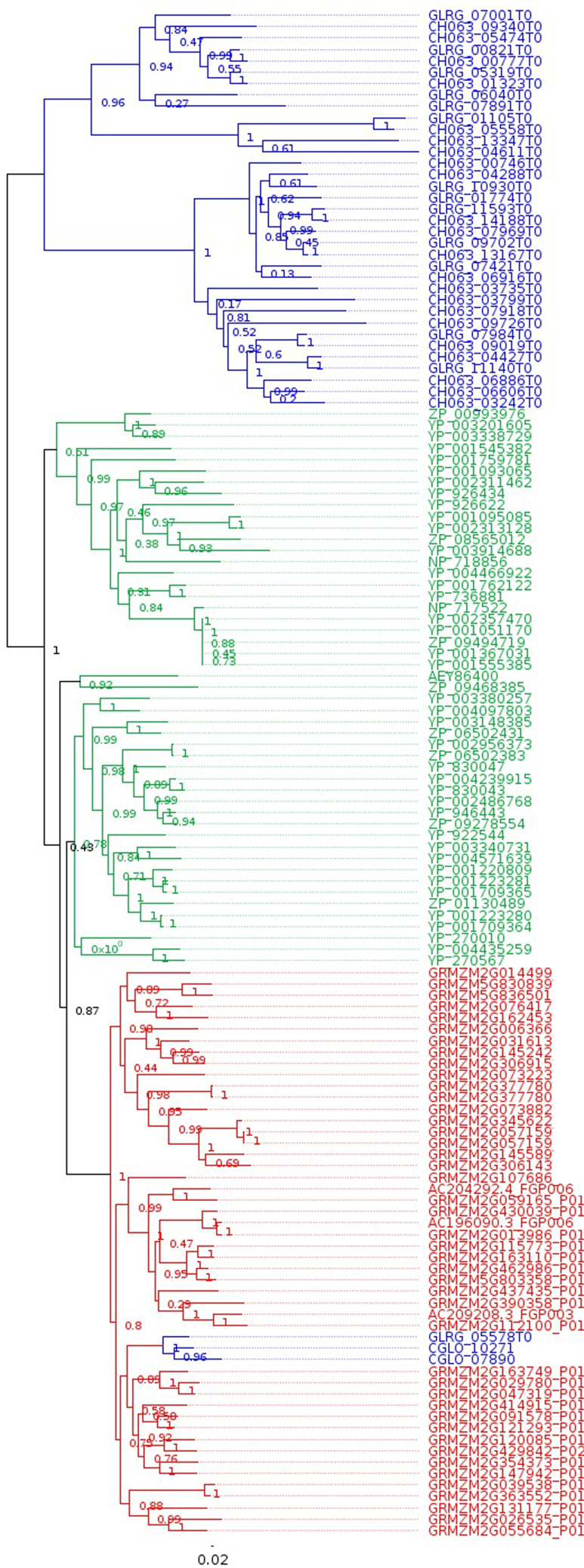
**

CPLS’s

**Figure S3**. Tree of all subtilisins found in maize and two species of *Colletotrichum*, including representatives from bacterial phyla. The accession numbers of genome project are shown for maize and *Colletotrichum* sequences and GenBank accession number for bacteria. *Zea mays* (GRMZM2G or AC) colored in red, two species of *Colletotrichum* (*C. graminicola* GLRG and *C. higginsianum* CH) are colored in blue and subtilisins from bacteria in green. Internal nodes are labeled with SH-like support values.


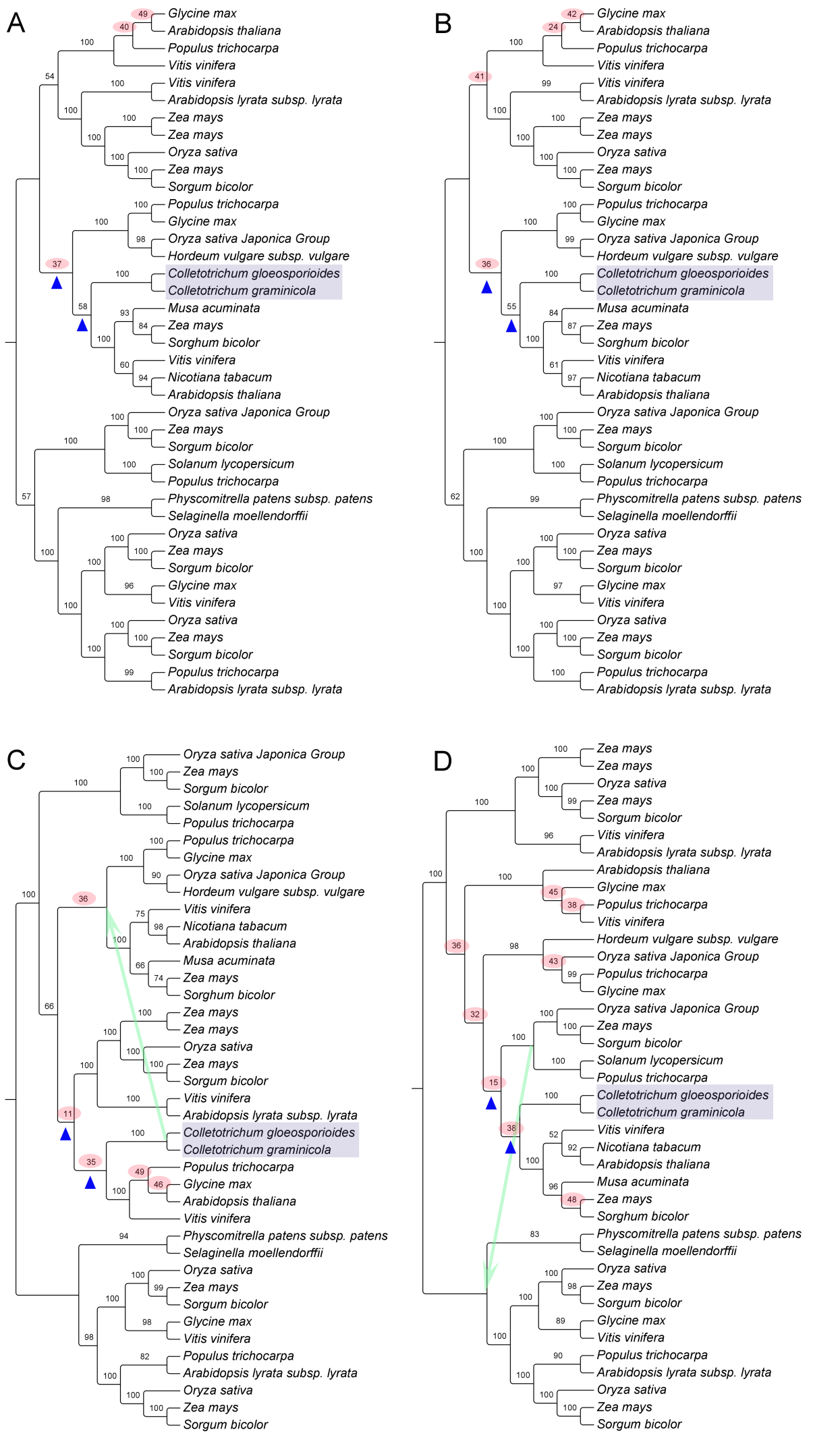


**Figure S4**. Trees reconstructed in PhyML (100 bootstrap) with the same dataset using four different alignment editing methods. **A**) Manually edited. **B**) Unedited. **C**) Automatially edited with the trimAl program. **D**) Automatically edited with the Gblocks program. The red circles highlight the bootstrap support values lower than 50%. Blue triangles indicate the support values for the key nodes representing the insertion of CPLSs in the plant lineage. The green arrows in C and D trees show the differences in the position of some clusters taking as a reference the topology of A and B trees. The dataset and the nomenclature are the same used in figure 3 of the main document. The original alignment was performed in MAFFT.


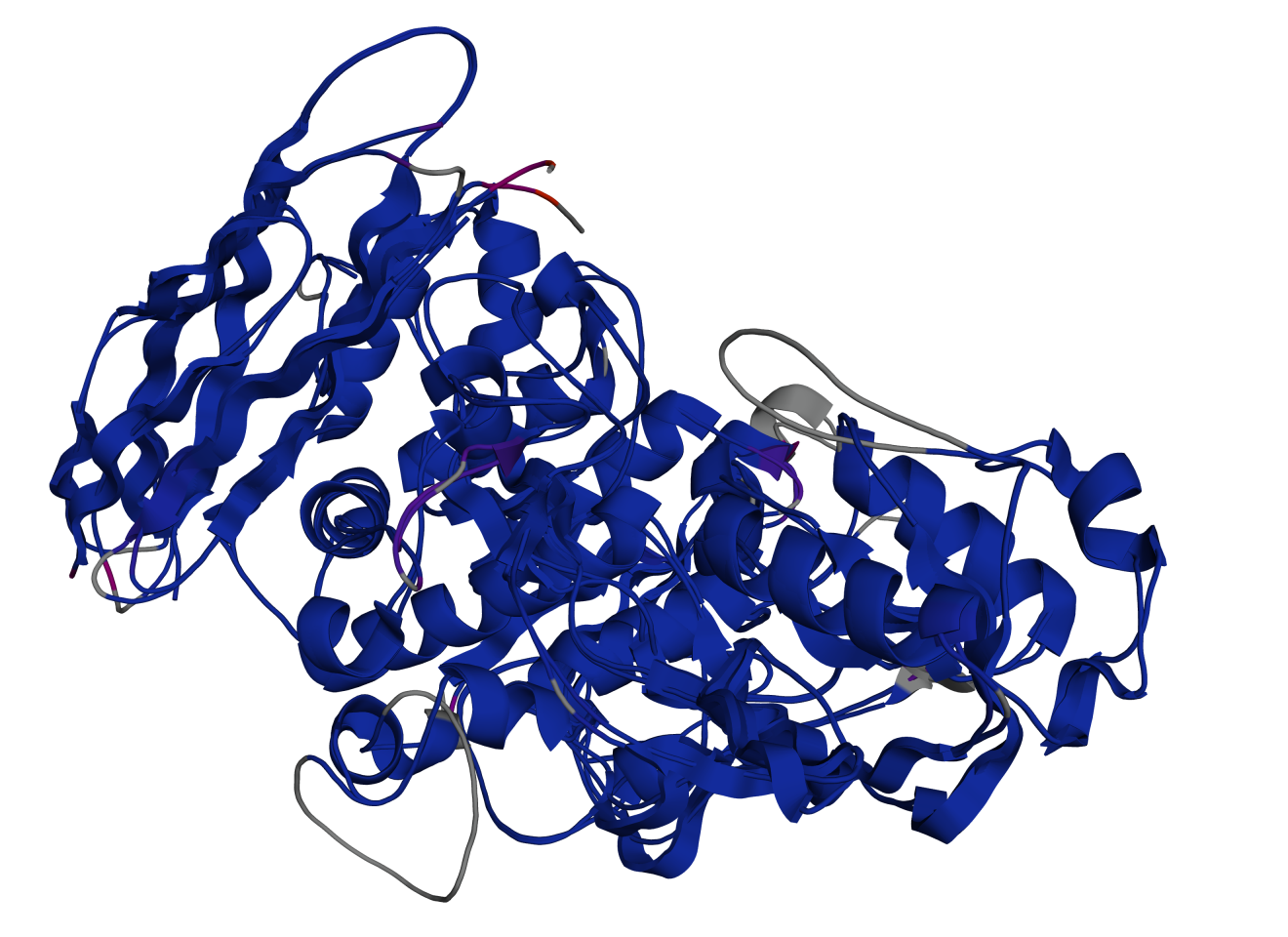


**Figure S5**. Structural alignment between SBT3 of maize and GLRG_05578 of *C. graminicola*. The color spectrum represents the values of pairwise RMSD, with blue specifying minimum values and red indicating maximum values. Gray sections are not aligned.

**
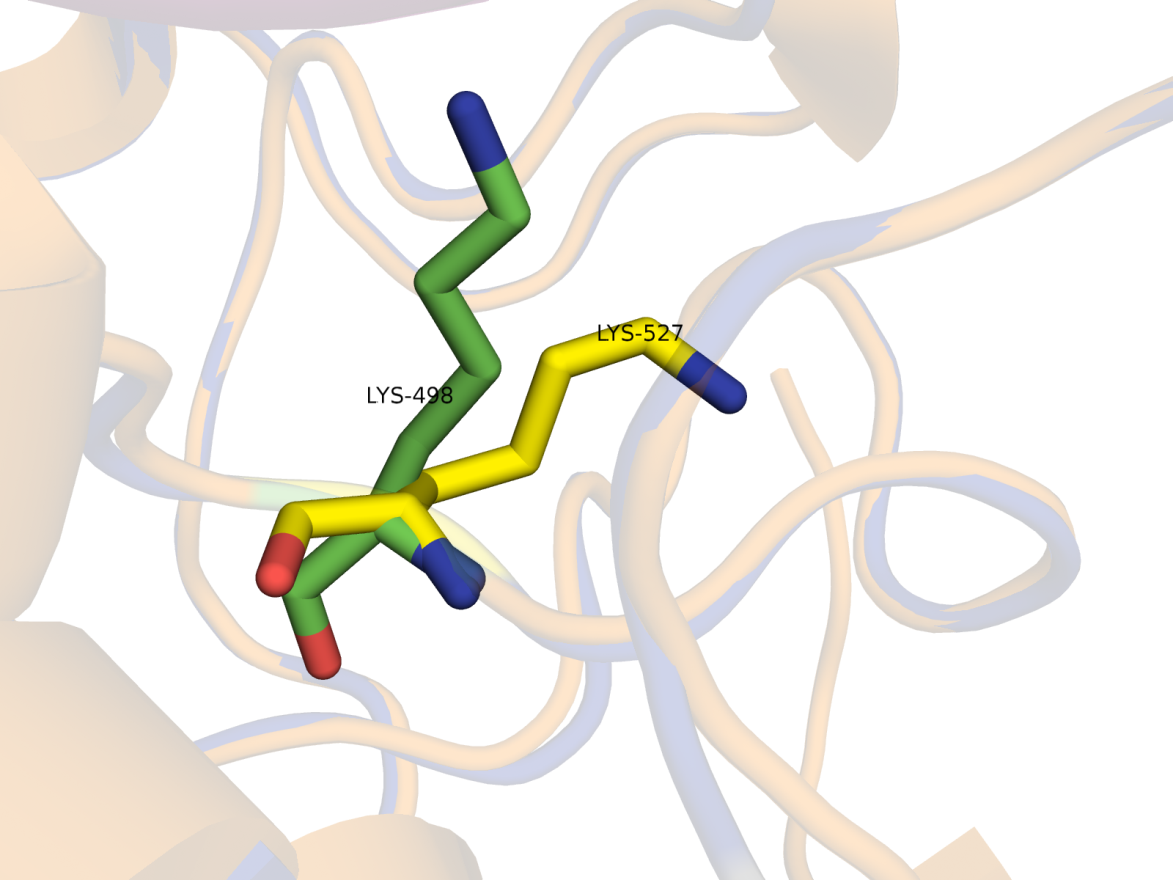
**

**Figure S6**.Structural alignment between residue Lys-498 of SBT3 (a putative stabilization site of the proteins to high temperature and alkalinity) and residue Lys-527 of CPLS GLRG_05578. Carbons atoms are represented in green and yellow respectively, oxygen atoms in red and nitrogen atoms in blue. Transparent cartoon representation of nearby structures is colored in blue for GLRG_05578 and in orange for SBT3.

A

**
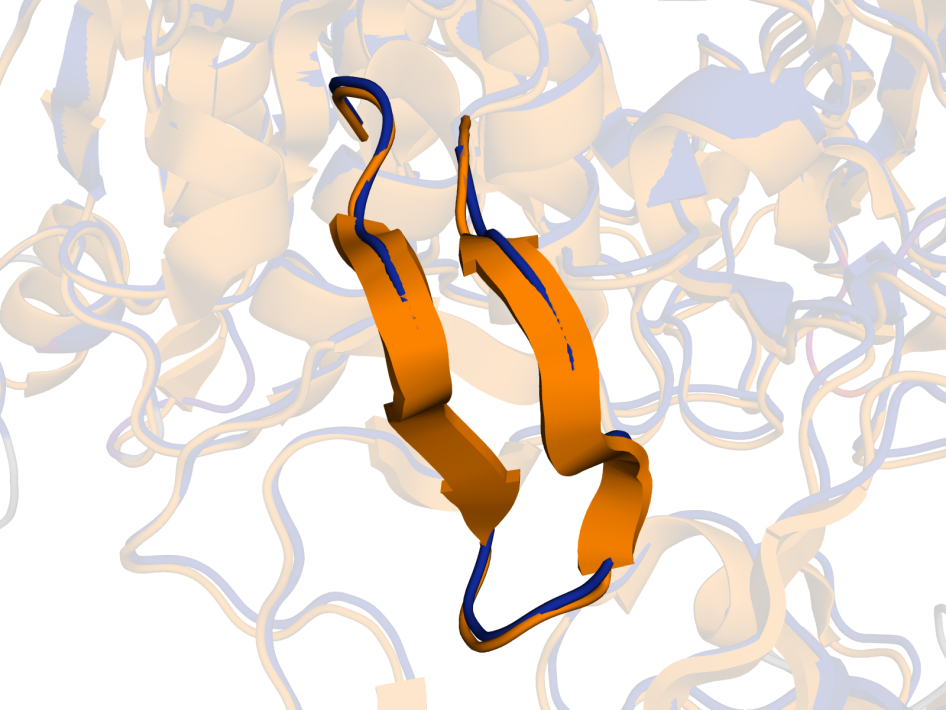
**

B


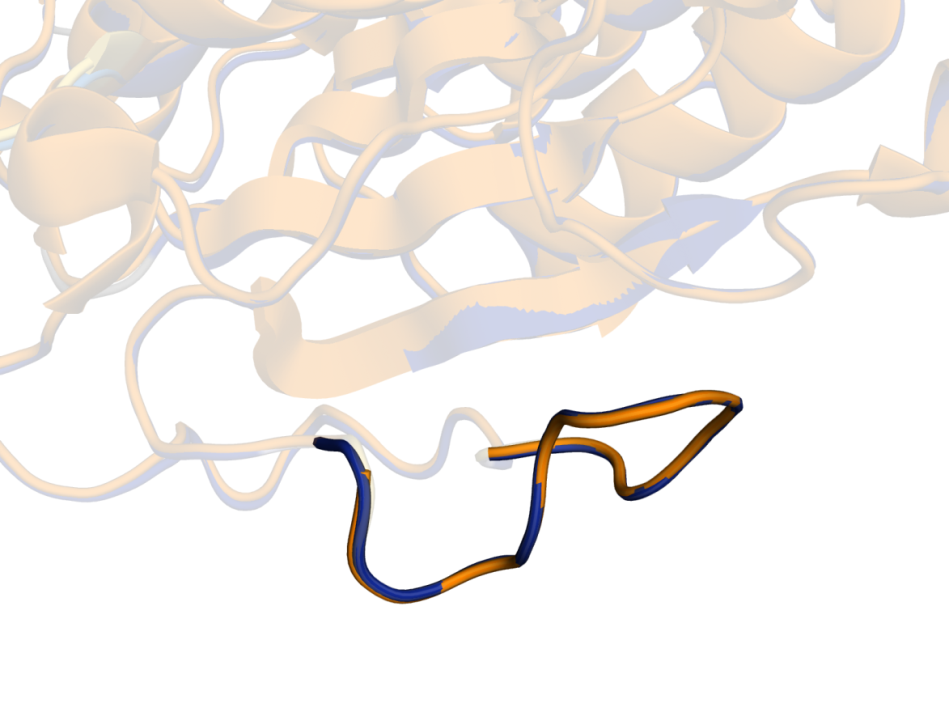


**Figure S7.A)** Structural alignment of SBT3 and GLRG_5578 in the Ca-1 region. **B)** Structural alignment of SBT3 and GLRG_5578 in the Ca-2 region. SBT3 protein is colored in orange and GLRG_5578 protein is colored in blue.

**
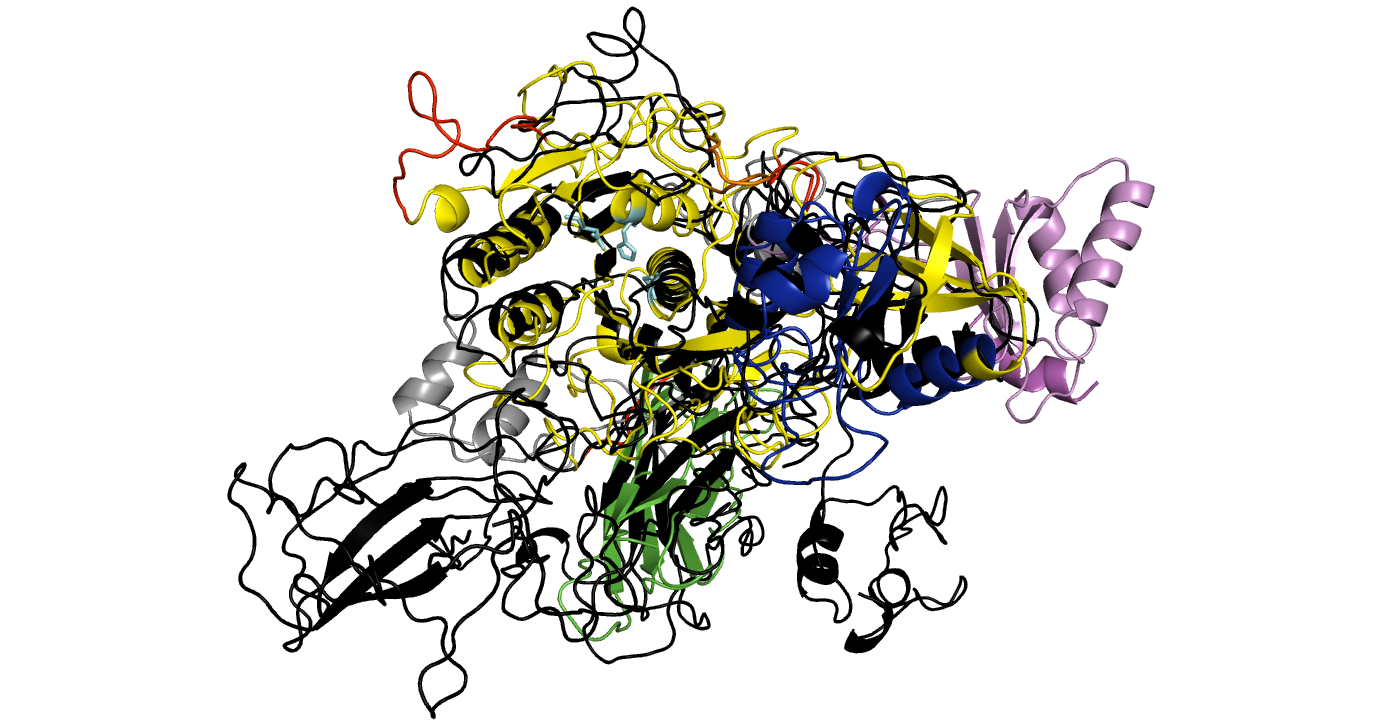
**

**Figure S8**. 3D alignment between the CPLS GLRG_05578 and GLRG_07421 of *C. graminicola.*The alignment emphasizes the structural differences between these two *C. graminicola* subtilisins. For GLRG_05578 peptidase s8 domain is colored in yellow, PA domain in blue and Fn III-like in green. The ß-hairpin like is colored in orange, the residues of the catalytic site are colored in cyan (and highlighted in stick view) and the putative sites of Ca^+^ replacement are in red. The signal peptide and I9 inhibitor are in pink and violet respectively. The gray parts have not assigned any characteristic. The structure of GLRG_07421 is entirely in black.
